# Supplementary material for: The emergence of scale-free fires in Australia
Source: iScience. 2023 Feb 13;26(3):106181. doi: 10.1016/j.isci.2023.106181 (PMC9988665; doi:10.1016/j.isci.2023.106181)
Supplement: Document S1. Figures S1–S4 [file mmc1.pdf]

iScience, Volume *26*

Supplemental information

The emergence of scale-free fires in Australia

Giorgio Nicoletti, Leonardo Saravia, Fernando Momo, Amos Maritan, and Samir Suweis

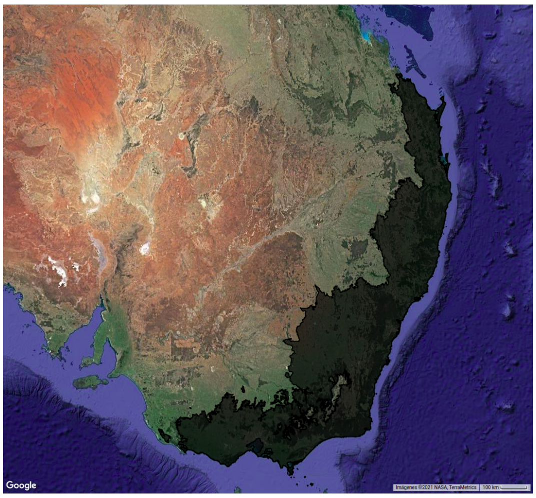

Figure S1: Region of study, related to STAR Methods. The shaded area represents the region of study encompassing the East and Southeast temperate broadleaf and mixed forests of continental Australia.

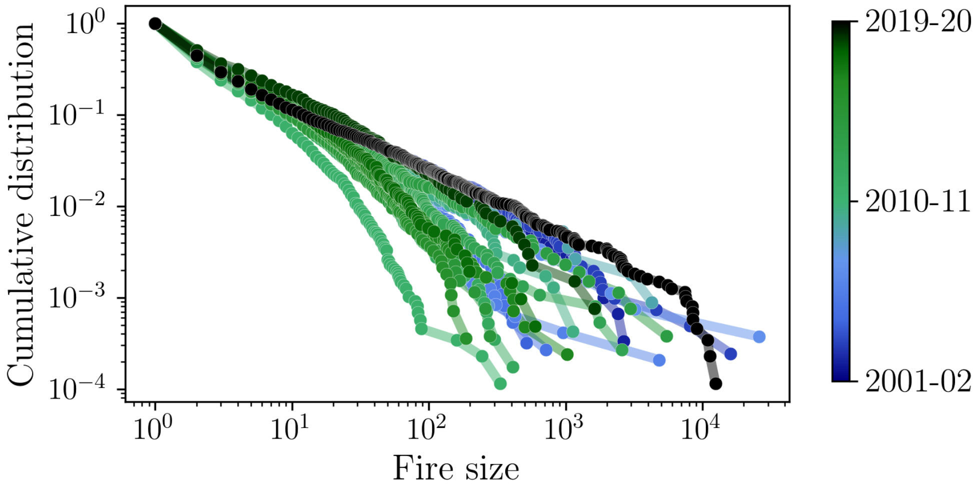

Figure S2: Comparison of yearly distributions of fire sizes from 2001-2002 to 2019-2002, related to Figure 1.

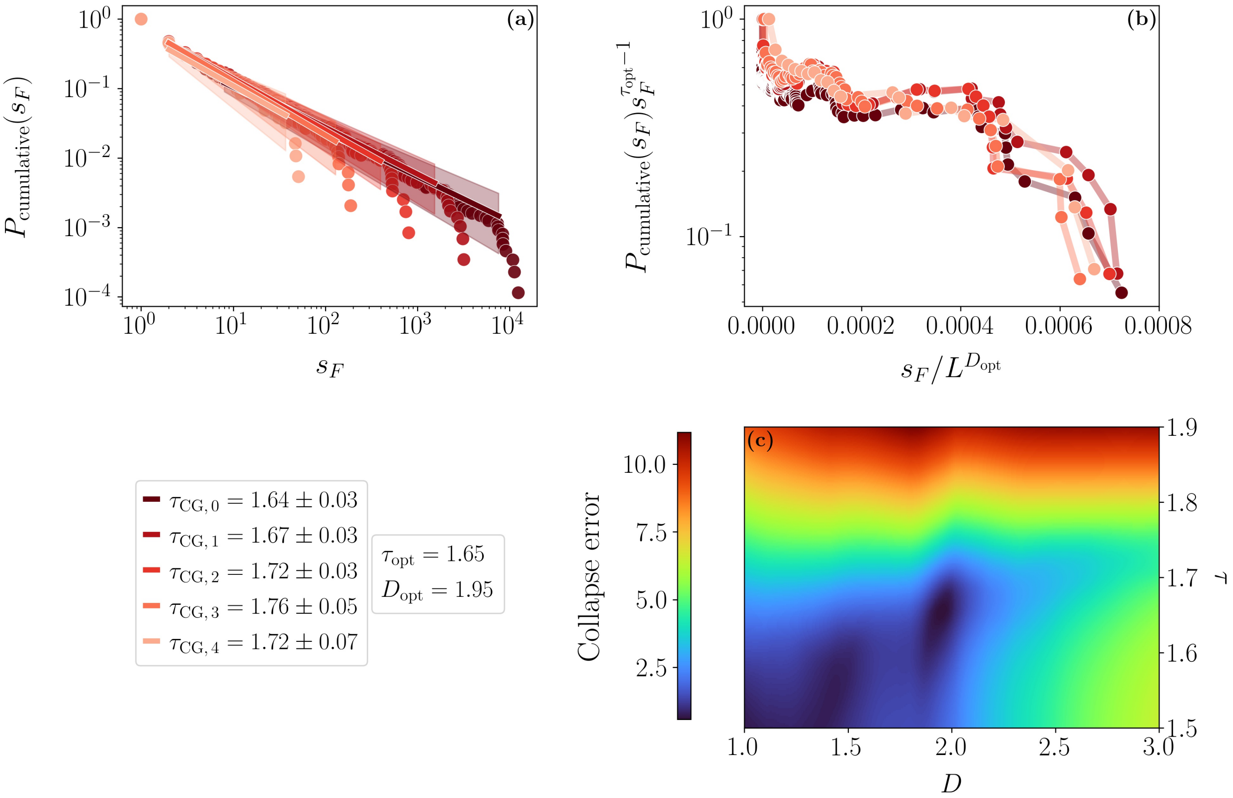

Figure S3: Invariance under coarse-graining and data collapse, related to Figure 3 and STAR Methods. (a) The cumulative distribution of the fire sizes during 2019-2020 along the coarse-graining transformations, with the best power-law fit obtained by maximum-likelihood[1]. The exponents remain compatible at different CG steps. Notice that the different τ reported here those of the fire size distribution, not of the cumulative distribution. (b) The cumulative distributions of the data at different CG steps collapse into the same curve, once appropriately rescaled, as predicted by finite-size scaling. In order to appreciate the quality of the collapse, notice the different units in the vertical axis with respect to panel (a). (c) The two parameters τ and D that are needed to collapse the cumulative distributions of the fire sizes can be chosen so that the collapse error will be minimal[2]. As expected, we find $\tau_{\mathrm{opt}} \approx 1.65$, which is compatible with the fitted exponent.

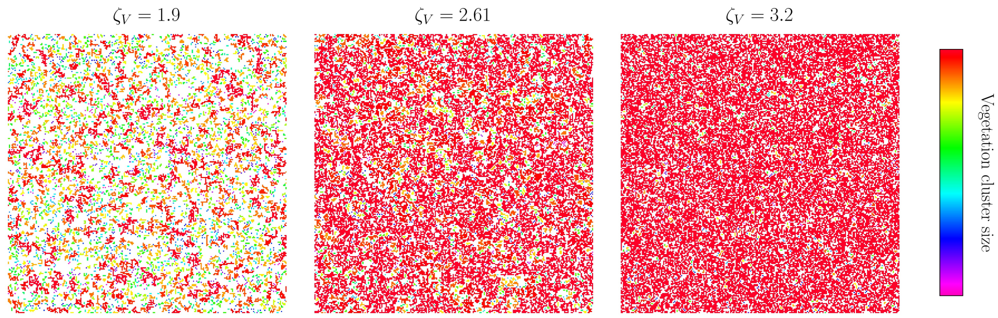

Figure S4: Stationary configurations of the vegetation contact process, related to Figure 4. Different colors represent clusters of different sizes in a 250×250 2-dimensional lattice and t different values of ζ_V. Notice how at the percolation threshold a system-size cluster appears.

References

1. Clauset, A., Shalizi, C. R., and Newman, M. E. (2009). Power-law distributions in empirical data. SIAM review *51*, 661–703.

2. Bhattacharjee, S. M., and Seno, F. (2001). A measure of data collapse for scaling. Journal of Physics A: Mathematical and General *34*, 6375.
